# The Mechanisms Behind Rapid Antidepressant Effects of Ketamine: A Systematic Review With a Focus on Molecular Neuroplasticity

**DOI:** 10.3389/fpsyt.2022.860882

**Published:** 2022-04-25

**Authors:** Melody J. Y. Kang, Emily Hawken, Gustavo Hector Vazquez

**Affiliations:** ^1^Center of Neuroscience Studies (CNS), Queen’s University, Kingston, ON, Canada; ^2^Department of Psychiatry, Queen’s University School of Medicine, Kingston, ON, Canada; ^3^Providence Care Hospital, Kingston, ON, Canada

**Keywords:** bipolar disorder, ketamine, major depressive disorder, mechanism of action, neuroplasticity, treatment-resistant depression, rapid antidepressant effects

## Abstract

The mechanism of action underlying ketamine’s rapid antidepressant effects in patients with depression, both suffering from major depressive disorder (MDD) and bipolar disorder (BD), including treatment resistant depression (TRD), remains unclear. Of the many speculated routes that ketamine may act through, restoring deficits in neuroplasticity may be the most parsimonious mechanism in both human patients and preclinical models of depression. Here, we conducted a literature search using PubMed for any reports of ketamine inducing neuroplasticity relevant to depression, to identify cellular and molecular events, relevant to neuroplasticity, immediately observed with rapid mood improvements in humans or antidepressant-like effects in animals. After screening reports using our inclusion/exclusion criteria, 139 publications with data from cell cultures, animal models, and patients with BD or MDD were included (registered on PROSPERO, ID: CRD42019123346). We found accumulating evidence to support that ketamine induces an increase in molecules involved in modulating neuroplasticity, and that these changes are paired with rapid antidepressant effects. Molecules or complexes of high interest include glutamate, AMPA receptors (AMPAR), mTOR, BDNF/TrkB, VGF, eEF2K, p70S6K, GSK-3, IGF2, Erk, and microRNAs. In summary, these studies suggest a robust relationship between improvements in mood, and ketamine-induced increases in molecular neuroplasticity, particularly regarding intracellular signaling molecules.

## Introduction

Depressive Disorders affect 280 million people globally, being the leading cause of disability worldwide (1). One widely accepted neurobiological theory for this highly prevalent mental disorder is the monoamine hypothesis of depression, developed over 50 years ago (2), suggesting that serotonin, norepinephrine, and dopamine deficiencies were responsible for the occurrence of depressive symptoms. Since then, all antidepressant drugs, such as selective serotonin reuptake inhibitors, monoamine oxidase inhibitors, and tricyclic antidepressants have targeted this system to provide relief. While effective for some, remission rates remain low with a delayed onset of clinical efficacy, and up to 46% of patients do not respond effectively to available treatments—resulting in treatment-resistant depression (TRD) (3). One accepted definition of TRD is when patients exhibit inadequate responses to two or more antidepressants after adequate dosing and duration (3). However, in practice, many patients with TRD have exhausted all available treatments, yet struggle to find symptom relief. This highlights the complexity of this disorder as well as the deficiency of evidence regarding its pathogenesis and methods of treatment.

The neuroplasticity hypothesis of depression is a recent neurobiological theory of major depressive disorder (MDD). Neuroplasticity is a phenomenon characterized by neuronal adaptation, i.e., the brain’s ability to reorganize itself when facing an internal or external stimulus (4–8). MDD is strongly linked to abnormalities in neuroplasticity shown through neuroimaging and pharmacological studies (9, 10). When referring to neuroplasticity, this term generally encompasses three large categories; structural, molecular, and functional neuroplasticity (11). Changes in volumes of brain regions, phenotypical changes in neuronal cell types, and differing levels of biomarkers involved in neuroplasticity signaling cascades are all observed in both patients with depressive symptoms and animal models of depression, compared to healthy controls (9, 10). Furthermore, traditional antidepressant medications that target the monoamine hypothesis of depression show downstream effects of improved neuroplasticity in patients, and rescued neuroplasticity in animal models of depression (11–13). Through these observations, the deficit of neuroplasticity has been elucidated as a potential target in developing novel therapeutics for the pathology of depression.

Ketamine, an N-methyl-D-aspartate receptor antagonist within the glutamatergic system, was first approved by the U.S. Food and Drug Administration (FDA) as an anesthetic in 1970. In recent years, many studies have explored its antidepressant properties and demonstrated its potential for patients with MDD and TRD. Following these studies, the FDA approved the isomer (s)-ketamine, also known as esketamine, as the first glutamatergic antidepressant in the form of an intranasal spray called Spravato, in 2019 (14). Unlike currently available pharmacological antidepressants, ketamine elicits its effects as fast as 1 h following administration and is sustained for up to 1–2 weeks (15, 16). It also shows high response and remission rates at approximately 60–70% and 30%, respectively (17–20). Additionally, ketamine also affects several domains of neuroplasticity including molecular biomarkers, cellular structures, and regional brain volumes, raising the question about the underlying mechanisms of action mediating ketamine’s rapid clinical antidepressant effects.

The exact relationship between the pathology of depression, deficits in neuroplasticity, and ketamine’s rapid antidepressant effect remain poorly understood. Current results of the literature seem to fall into two different categories of mechanisms that converge to enact ketamine’s antidepressant effect: molecular neuroplasticity for its immediate effects (minutes to hours), and structural neuroplasticity for its sustained effects (days). Molecular plasticity affected by ketamine include changes in receptors, primary and secondary effectors, proteins involved in signaling cascades, neurotransmitters, and microRNAs. These changes are observed immediately post infusion in both humans and animal models and seem to precede any other structural or functional plasticity markers observed. Here we have summarized the broad range of ketamine’s effects on neuroplasticity in the context of depression, with a specific focus on the immediate *molecular cascades* that may mediate ketamine’s rapid antidepressant effects. In conjunction, we postulate that deficits or alterations in molecular neuroplasticity may constitute pathological markers of depression and that ketamine might serve to restore plasticity to induce its rapid antidepressant effect.

## Methods

Using Preferred Reporting Items for Systematic Review and Meta-Analysis (PRISMA) guidelines (21), the search for this systematic review was conducted through January 2022 and registered on PROSPERO (ID: CRD42019123346). The database PubMed was used to identify peer-reviewed literature regarding ketamine and neuroplasticity in both preclinical and clinical trials, with or without controls or randomization. Our search included open-label studies, as well as citations from reviews for additional papers to include.

Search terms were the following: “ketamine” and “neuroplasticity, dendritic spines, spinogenesis, synaptogenesis, dendritic arbor, synapse, neurogenesis, neuroregeneration, neurotransmission, BDNF, neurotrophic, neuronal plasticity, nerve growth factor, mTOR, synaptic plasticity, functional plasticity, glutamate, cognitive flexibility, and GSK-3.” The search strategy was reviewed by all authors and reports were reviewed by two investigators independently (M.JY.K and E.R.H) to verify correct filtering and sampling process. Screening selected titles and abstracts, as well as full article eligibility assessments were completed by the same two investigators independently, with continuation to full texts of reports. Inclusion criteria included reports that were peer-reviewed and published that evaluated ketamine’s neuroplastic nature either *in vivo*, *in vitro*, both in animals and humans, in an antidepressant context, and written in English. Exclusion criteria included studies regarding schizophrenia or any other disorder not relevant to bipolar disorder, MDD or TRD, chart reviews, and reports lacking data on significant changes. In addition, any studies regarding structural or functional (electrophysiology, connectivity, potentiation) neuroplastic changes post-ketamine were excluded as it was out of the scope of the current review. Data was sought for study design, sample size, route of administration or exposure to ketamine, measures of improvement, main results, and fundamental mechanism of neuroplasticity, conclusions, and limitations.

**FIGURE 1:**
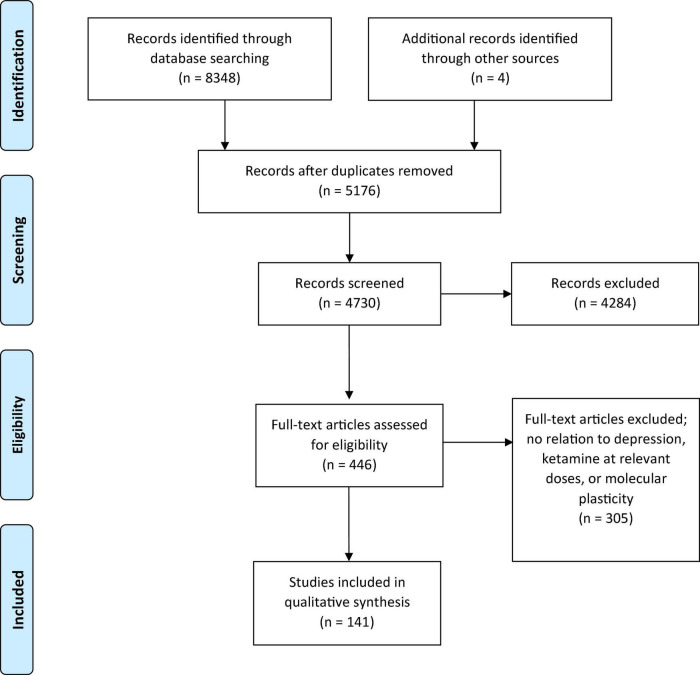
Schematic representation of PRISMA methods utilized for this systematic review.

During the first search, 8,352 reports were identified, and after removal of duplicates, 5,176 papers were considered. Another 4,730 papers were excluded during the title and abstract screening process for lack of relevance, with a remainder of 446 reports for full text review. This process led to another exclusion of 305 reports for reasons including manuscripts only studying ketamine at anesthetic doses, results mainly surrounding ketamine’s metabolite hydroxynorketamine, results only surrounding ketamine-induced behavioral changes, main pathologies explored were not MDD or TRD, timelines explored were only long-term and chronic, and results were surrounding functional or structural plasticity which was out of scope for our current review which focuses on molecular plasticity, resulting in a final number of 141. The results from these reports are all included in this systematic analysis (Figure 1).

## Results

### Glutamate/Gamma-Aminobutyric Acid Neurotransmission and Its Receptors Are Highly Affected by Ketamine

Glutamate is the main excitatory neurotransmitter in the brain and has functionality in over half of the total neuronal synapses. Differing levels of glutamate has been reported in healthy controls vs. patients with depression (22), as well as in post-mortem studies of suicide victims (23). The transmission of glutamate, its precursor glutamine, as well as the main inhibitory neurotransmitter gamma-aminobutyric acid (GABA), has been explored as a part of ketamine’s mechanism of action.

After exposure to ketamine, a glutamate burst in the synapse is observed, and this is thought to be caused by the binding of ketamine to inhibitory interneurons and thereby enhancing the actions of pyramidal excitatory neurons (24–28). Downstream signaling molecules of this process, such as neuregulin-1, has shown to be downregulated post ketamine administration in parvalbumin-expressing (PV) interneurons, resulting in cortical disinhibition (29). In addition, this glutamate burst is not observed with traditional antidepressants (24–28, 30), though it seems to be dependent on specific synapses and is not globally observed (31). Some studies suggest that this burst may also be sex-dependent (32).

Fluctuating levels of glutamate, glutamine, and GABA following ketamine depend on brain region, timing of measurement, and species. Clinical studies offer varied results. Acute increases in levels of glutamate, glutamine, and GABA have been found in the medial prefrontal cortex (mPFC) of patients with MDD post-ketamine, with GABA levels decreasing after 3–4 h (33, 34). This reduction is maintained for up to 24 h post-ketamine (35). Another study, published by the same group, found the opposite result in the ventro-mPFC, with glutamine and glutamate (Glx) levels decreasing post ketamine, with lower mean Glx levels associated with a better antidepressant response (36). They postulate the reason for their opposite result to be the existence of a placebo group in the latter trial (36). Glx cycling has also shown to contribute to ketamine’s mechanism in the mPFC (37) and the pregenual anterior cingulate cortex (pgACC) (38, 39). The pgACC of healthy controls and patients with MDD demonstrated no changes in glutamate levels, but a significant increase in the ratio of glutamine/glutamate, after 24 h (38, 39). Supporting this, ketamine induced an increase in prefrontal glutamate release in humans, measured by ^13^C-glutamine labeling of glutamine, which were more prominent than ^13^C-glutamate (40). This suggests an increased production of glutamine, but not glutamate post-ketamine. The above clinical studies employed magnetic resonance spectroscopy (MRS) and used the most observed subanesthetic dose of 0.5 mg/kg of ketamine, with the exception of the ^13^C-MRS study, which administered a 0.23 mg/kg bolus followed by a 0.58 mg/kg infusion.

Similar fluctuation patterns are observed in the prefrontal cortex (PFC) and hippocampus of rats (41–44), though one study showed decreased levels of Glx in the mouse hippocampus after 14 h, and increased GABA levels 72 h after ketamine exposure (45). Receptor subunit alterations are also observed. Hippocampal protein levels of GABA_*A*_ receptor subunit α1 significantly decreases acutely after ketamine in mice (45). Levels of GAD67, a GABA synthetic enzyme, are restored with ketamine administration in stressed models of mice (46), though another found reduced levels of GAD67 post-ketamine in the rat PFC (47). One study found that ketamine’s mechanism may involve changes in G-protein signaling regulators, such as RGS4, in the glutamatergic synapse (48). The rapid antidepressant effects may result directly from the alterations or restorations of these glutamatergic neurotransmitters, neuromodulatory regulators, or from a trigger of receptor activated signaling cascades from the increase of glutamine and glutamate. The nature of these changes in glutamate, glutamine, or GABA levels is still not defined. Ketamine doses used in animal studies most commonly range from 10 to 30 mg/kg, a comparable subanesthetic dose used frequently in rodent and animal studies.

AMPA receptors (AMPAR), one of three glutamatergic receptors, mediate most of the synaptic transmission that occurs in the brain and is likely integral in ketamine’s mechanism of action. AMPARs are located on post-synaptic neurons, and are comprised of GluA1, GluA2, GluA3, and GluA4 subunits (49, 50). Under normal conditions, the consequences of AMPAR activation result in downstream effects including fast excitatory synaptic transmission and increased signaling cascades integral to synaptic plasticity (49, 51). AMPARs and their receptor subunits have been implicated as pivotal factors in the control of mood as well as the pathophysiology and treatment of depression (52). Observed increases in glutamate is presumed to enhance the activation of AMPARs, to carry out ketamine’s antidepressant effects.

Many studies support activity at AMPARs as significant contributions to ketamine’s antidepressant effect. For example, when ketamine is administered at a dose below the threshold of response, an AMPA agonist induces an antidepressant-like effect in a rat model of depression (53). Even when the AMPA agonist is administered alone, similar responses are observed in rats, followed by increased downstream molecular effects, such as increased mTOR activity and BDNF levels (54). Ketamine also upregulates the mRNA of AMPAR subunits 1.5–2-fold (55). When an AMPAR antagonist, NBQX, is administered, ketamine’s rapid behavioral antidepressant-like effects are blocked (54, 56–58). NBQX is shown to inhibit ketamine-induced glutamate and GABA release in the mPFC of mice (58). In addition, ketamine-induced increases in mTOR signaling and protein levels in the hippocampus and prefrontal cortex are blocked by AMPAR antagonists (54, 59, 60). The inverse is true as well—downstream signaling inhibition reduces AMPAR activation and prevents the effects of ketamine (61). As expected, ketamine administration also increases the AMPA to NMDA receptor ratio transiently, a marker of increased activity at AMPARs, in the hippocampus of rat models of anxiety (62). These observations are not limited to *in vivo* studies, as AMPA antagonists abolish all spontaneous activity of ketamine in cultured neurons of the dorsal raphe nucleus (61).

There is some debate on whether ketamine administration phosphorylates subunits of the AMPAR, as a method of enhancing AMPA activation. Ketamine administration has shown to induce phosphorylation of the AMPA GluA1 subunit on the post synaptic terminal (63, 64). In addition, animals with a knockout of this phosphorylation site were unable to produce ketamine’s electrophysiological and rapid antidepressant-like effects (63). This suggests that phosphorylation of GluA1 is a requirement for a therapeutic response. However, a separate study observed ketamine inducing significantly lower levels of rodent hippocampal phosphorylated GluA1 (57). This effect was inhibited by AMPA antagonists (56). Opposite to results observed in GluA1, a stress paradigm in a rat model of depression increased hippocampal GluA2 phosphorylation levels, which was partially reduced by acute ketamine administration (65), though some studies showed opposite results where ketamine upregulated GluA2 subunits in both the mPFC and hippocampus of rodents (45, 64). In *in vitro* studies of human induced pluripotent stem cell (iPSC)-derived dopaminergic neurons, GluA1 and GluA2 increases were observed (66), though this was specific to dendrites. Only GluA2 and not GluA1 upregulation was observed in the soma (66). Levels of subunit GluA3 was significantly reduced in the hippocampus of vehicle mice treated with s-ketamine (45). Selective upregulation of subunit expression specific to regions of the neuron and brain may contribute to ketamine’s mechanism.

**FIGURE 2:**
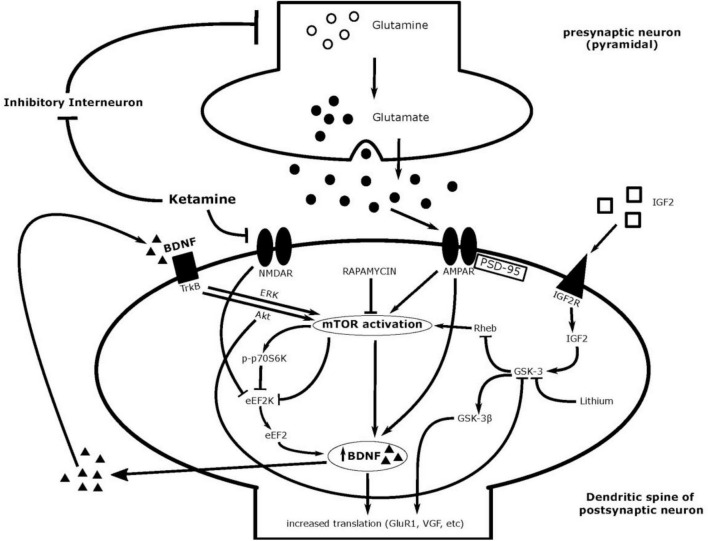
Ketamine’s postulated mechanism of action and associated molecules.

Along with receptors for glutamate, glutamate transporters are affected by ketamine administration. Stressed mice show decreased levels of excitatory amino acid transporters (EAAT) 2 and 3 and increased extracellular concentrations of glutamate—exhibiting improper glutamate transmission (67). Ketamine administration show restoration of EAAT levels coupled with behavioral antidepressant-like effects (67). Ketamine as an augmentative compound administered with guanosine diminished depressive-like behavior in rodent models of depression, and normalized decreased levels of hippocampal levels of glutamate transporter-1 (GLT-1) (68–70). However, another study showed decreased levels of GLT-1 with ketamine exposure in the mPFC (64). This suggests differential mechanisms of ketamine in the hippocampus and mPFC of animal models and are summarized in Table 1, and illustrated in Figure 2.

**TABLE 1:** Molecular changes observed after ketamine administration in preclinical (cell cultures, animals), and clinical studies.

	*In vitro*	Animals, *in vivo*										Humans			
		Brain region									Blood	Neuroimaging		Blood	
		PFC	HPC	AMY	DG	DRN	PAG	NAc	VTA	SN	Serum	mPFC	pgACC	Serum	Plasma
Glutamate		↑ (43)	↑ (43) ↓ (45)									↑ (33, 34) ↓ (36)	− (38, 39)		
Glutamine			↓ (45)									↑ (33, 34, 40) ↓ (36)			
Glx cycling		↑ (41)										↑ (37)	↑ (38, 39)		
GABA		↓ (43)	↓ (43) ↑ (45)									↑ (33) ↓ at 3–4 h (34) − at 24 h (35)			
GAD67		↓ (43) ↑ (46)	↓ (43)												
Neuregulin-1	↓ (29)	↓ (43)	↓ (43)												
RGS4	↓ (48)														
NMDA GluN2A					↓ (155)										
NMDA GluN2B		↑ (64)			↑ (155)										
NMDA GluN1															
NMDA GluN3					− (155)										
AMPAR	mRNA ↑ (55)				− (155)										
AMPAR/ NMDAR ratio			↑ (62)												
AMPA GluA1	*p*↑ (63, 64) ↑ (66)	↑ (73, 75)	*p*↓ (57) ↑ (76, 149)	↓ (82)											
AMPA GluA2	↑ (66)	↑ (64)	↑ (64) *p*↓ (65)												
AMPA GluA3			↓ (45)												
AMPA GluA4					↓ (83)										
EAAT2 and EAAT3			↑ (67)												
GLT-1		↓ (64)	↑ (68–70)												
mTOR		↑ (73) ↓ female only (32)													
p-mTOR			↑ (74)			↑ (61)									
p4E-BP		↑ (73)													
p70S6K		↑ (73)													
ERK		↑ (73)													
ERK44 and ERK42				↑ (82)											
Akt		↑ (73)													
PSD-95		↑ (73, 75)	↑ (76, 108), (154)												
p-PSD-95			↓ (153)												
Egr-1															
Rheb	↑ (88)														
BDNF	↑ (60, 114, 118)	↑ (102, 109), (115, 120), (121)	↑ (104–110), (115, 121), (122)	↑ (102, 111), (121)	↑ (109)		↑ (115)				↑ (112)			↑ (126) − (129)	↑(127, 128) − (129–133)
BDNF mRNA	↑ (118)		↑ Female only (158), − (117)		↑ Female only (158)										
BDNF exon IV mRNA			↑ (118)												
BDNF gene		↑ (103)													
BDNF promotor IV															
HDAC		↓ (109)	↓ (109)												
ProBDNF			↓ (119)												
p-TrkB	↑ (164)	↑ (164)													
p-CREB	↑ (164)	↑ (164)	↓ (164)												
VGF		↑ (140)													
eEF2K															
p-eEF2K		↑ (143)	↓ (90)												
p-p70S6K	↑ (60, 88)	↑ (59, 146)	↑ (88, 146)	↑ (146)				↑ (146)	↑ (146)	↑ (146)					
GSK-3		↓ (109)	↓ (109, 150)		↓ (109)										
GSK-3β		↑ (151, 152)													
IGF2	↑ (157)														
VEGF			↓ (169)												− (131)
VEGFA															↑ (170)
VEGF mRNA															↑ (170)
miR-29b-3p		↑ (171)													
GRM4		↓ (171)													
miR-206			↓ (172)												
miR-9-5p	↑ (173)		↑ (173)												

*↑ Indicates increase, ↓ indicates decrease, − indicates no change, p means phosphorylation, superscripts correspond to reference list.*

### Ketamine Induces a Net Increase in the Activation of the Mechanistic Target of Rapamycin (mTOR) Signaling Pathway

Recent studies outline the mechanistic target of rapamycin (mTOR) as one of the kinases involved in ketamine’s mechanism of action. mTOR is triggered by both the activation of AMPARs and the antagonism of NMDA receptors from ketamine binding. This protein kinase has many upstream and downstream effectors and is known as a master regulator of cell growth that responds to various physiological cues such as amino acids, stress, growth factors, and metabolism (71). mTOR combines with several different proteins to form a complex which function to execute cell survival, maturation, and maintenance (71). Knockdown of mTOR has previously evoked depressive-like states in mice (72) and rapamycin, an mTOR inhibitor, has allowed for the investigation of ketamine’s role in the mTOR signaling pathway.

Ketamine administration rapidly activates the mTOR pathway in animal models, and consequently increases levels of associated signaling proteins dose-dependently, such as phospho- eukaryotic initiation factor 4E binding protein 1 (p4E-BP), p70S6 kinase (p70S6K), extracellular signal-regulated kinase (ERK), and Akt (also named protein kinase B, PKB), an effect that is sustained for at least 72 h (73). Levels of phosphorylated mTOR increase in rat hippocampus and dorsal raphe nucleus post ketamine exposure (61, 74). Blocking the mTOR pathway with rapamycin results in inhibition of ketamine’s behavioral and molecular effects (73, 75) including increases in synaptic proteins such as postsynaptic density protein 95 (PSD-95), and GluA1 in the PFC (73, 75) and hippocampus (76). Ketamine-induced increases in PSD-95 were also observed in the somatosensory cortex of mice (77). These molecular changes may be very specific to brain region, as the ventral hippocampus of mice exhibit greater upregulations of proteins in the mTOR pathway, than the dorsal hippocampus (78). Pre-treatment with ketamine also prevents reductions of PSD-95, GluA1, and synapsin induced by models of depression (79–81). Ketamine also reverses stressed-induced changes in levels of GluA1 in the amygdala (82), hippocampus (76), and levels of GluA4 in the dentate gyrus (83).

*In vivo* deletion of GluN2B (NR2B), a subunit of the glutamatergic NMDA receptor increases mTOR signaling and mimics ketamine’s behavioral antidepressant-like effects in mice (84, 85), though one study found increases in GluN2B in the rat mPFC after ketamine exposure (64). Interestingly, deletion of another NMDAR subunit, GluN2C, fully preserves ketamine’s antidepressant-like effects in mice (86), suggesting only selective NMDA isomers are required in this mechanism.

An upstream G-protein, Ras homolog enriched in brain (Rheb), is involved in the regulation mTOR activity (87). Rheb, when bound to glycolytic enzyme glyceraldehyde 3-phosphate dehydrogenase (GAPDH) and therefore sequestered, inhibits mTOR activation. Conversely, it is implied that when Rheb-GAPDH binding is blocked, mTOR activation will increase and potential antidepressant mechanisms can ensue (88). In the presence of ketamine, levels of Rheb are significantly increased in cell culture, which is presumed to be closely associated with the increased levels of mTOR activity (88). When NMDA receptors are concomitantly activated, Rheb levels decrease, supporting NMDAR antagonism as part of ketamine’s mechanism of action (88).

The mTOR pathway largely initiates mRNA protein translation through eukaryotic initiation factor 4E-binding proteins (4E-BP), and the mTOR-4E-BP pathway has shown to be pivotal for synaptic plasticity (89, 90). Downstream signaling proteins 4E-BP1 and 4E-BP2 are required for antidepressant-like effects of ketamine and its metabolite, (2R,6R)-HNK, in rodents (89). Additionally, another regulator called extracellular signal-related kinases (ERK) has shown involvement in ketamine’s mechanism, which has previously demonstrated increased levels of phosphorylation in response to traditional antidepressants such as escitalopram, paroxetine, and tranylcypromine in rat hippocampal cultures (91). Ketamine reversed stress-induced decreases in levels of ERK 44 and 42 in the rat amygdala, reflective of increased mTOR activation (82). The mTOR and ERK pathway may only be specific to the mechanism of (S)-ketamine, as (R)-ketamine did not show changes in levels of phosphorylation in the mouse PFC (92).

A neurotrophin largely responsible for neuroplasticity in the brain is brain-derived neurotrophic factor (BDNF). BDNF is a marker of neuronal survival and growth and has been heavily associated with mood and antidepressant therapy (93, 94). BDNF is produced by mTOR activation, released into the synapse by the neuron, and stimulates its receptor, tropomyosin receptor kinase B (TrkB) on the same postsynaptic neuron (95, 96). This further stimulates mTOR activation and functions as a positive feedback loop (96).

Infusion of a BDNF neutralizing antibody, BDNF-knockout, and TrkB-knockout resulted in abolishment of ketamine’s behavioral antidepressant-like effects in animal models (90, 97–100). Similar results are observed with a TrkB antagonist (81, 101). Stress-induced decreases in levels of BDNF in the mouse hippocampus and the ventromedial prefrontal cortex is effectively restored with ketamine administration (102–105). Ketamine also acutely increases BDNF levels in the hippocampus and amygdala (106–111), dentate gyrus (109), and serum (112) of rodents. Administration of a TrkB inhibitor to the hippocampus blocks ketamine’s behavioral and molecular effects in a rat model of depression (81, 113). *In vitro* studies show similar effects. Primary neuronal cultures incubated with ketamine show increased release of BDNF at 15 min (60). Neuronal cultures isolated from the hippocampus of rat models of depression show increased levels of BDNF when the rats were treated with ketamine, as opposed to those that were not (114).

Interestingly, ketamine’s metabolite, (2R,6R)-HNK also upregulated BDNF levels in the rat ventrolateral periaqueductal gray, hippocampus, and mPFC (115). Infusion of (2R,6R)-HNK into the intramedial-mPFC or systemic administration induced antidepressant-like effects in mice (97). *In vitro* studies show that ketamine and (2R,6R)-HNK disrupts TrkB receptor interaction with a protein complex crucial for endocytosis, adaptor protein complex 2 (AP2M) (116). This suggests differential regulation mechanisms.

One study also showed differential effects of the R- and S- isomer requiring TrkB receptor activation involved in ketamine’s mechanism (92). Behavioral effects of R-ketamine required TrkB receptors, while S-ketamine did not (92). Though further research is required, these studies robustly support involvement of BDNF-TrkB signaling in ketamine’s mechanism.

Specific phases of upstream or downstream signaling of BDNF are also involved. One study showed ketamine-induced restoration of hippocampal dendritic trafficking of BDNF mRNA that was impaired by chronic mild stress in rats, though global levels of BDNF mRNA were not rescued (117). Significantly increased levels of BDNF promotor IV’s transcriptional activity has also been found post-ketamine (118). Ketamine decreases levels of a transcriptional regulator, histone deacetylase (HDAC), in the mouse striata, hippocampus, and PFC (109). However, knocking out histone deacetylase 5 (HDAC5) resulted in a dysregulated increase of BDNF, which abolished ketamine-induced increases (118).

ProBDNF, a BDNF precursor, is cleaved to produce mature BDNF (mBDNF) and this cleavage is assumed to be integral in producing antidepressant effects (119). The administration of ketamine increases mBDNF levels and decreases the ratio of proBDNF to mBDNF in stressed mice (119). When this cleavage is inhibited with a tissue plasminogen activator (tPA), mice show increased depressive-like symptoms (119). Additionally, the release of mBDNF post S-ketamine in the mouse mPFC was shown to be dependent on the expression of stress-responsive glucocorticoid receptor co-chaperone FKBP51, and FKBP51-knockout mice did not show antidepressant-like effects of ketamine (120). This suggests that this cleavage, and the expression of mBDNF, is integral in ketamine’s mechanism of action.

While most researchers agree on the importance of BDNF in ketamine’s rapid therapeutic effects, the exact role of BDNF is still contested. Measurement of BDNF protein levels induced by ketamine seems to vary as a function of time. Rats who were sacrificed immediately after administration had higher BDNF levels in the prefrontal cortex, amygdala, and hippocampus than those sacrificed 1 and 6 h post-administration (121). Acute administration of ketamine results in a spike of BDNF levels in the hippocampus (122), though chronic administration for 14 days results in BDNF hippocampal levels being unaffected (123, 124). BDNF protein levels seem to increase acutely, and decrease slowly, suggesting a trigger in homeostatic mechanisms to clear increased protein levels (121). However, increased BDNF is seemingly unnecessary for the sustained antidepressant effect. Though protein levels returned to baseline after 24 h, behavioral antidepressant-like responses were still intact in mice (90). In one study, ketamine was still able to manifest antidepressant-like effects in BDNF knockout mice unable to produce BDNF, suggesting that ketamine elicits its clinical effects via additional mechanisms (125).

Results of BDNF levels in humans show variability, and all clinical studies employed the common subanesthetic dose of 0.5 mg/kg, with the exception of two studies which included a range of 0.2–0.5 mg/kg. Ketamine-induced increases in BDNF are observed in serum after 1 week (126) and plasma after 230–240 min (127, 128). Depressive symptoms of patients correlated with BDNF plasma levels for up to 72 h (128), while one found no correlation with mood responses (129). Other studies have found no differences in BDNF levels in plasma at the same time points despite significant improvements in mood (129–133). In a genome-wide association study, single nucleotide polymorphisms as well as whole genes involved in BDNF-TrkB signaling were shown to be associated with rapid and sustained antidepressant effects in patients with TRD (134). Specific polymorphisms associated with BDNF, namely those with the Val/Val BDNF allele at rs65, were more likely to exhibit an antidepressant response (135) and an anti-suicidal response (136) to intravenous ketamine than those with the Val66Met polymorphism.

One critical limitation of using BDNF as a biomarker arises from the fact that quantification of BDNF is often conducted in animal models through brain tissue, and peripheral blood in humans. Robust animal data may not translate to humans in practice. For example, a robust increase in plasma BDNF from ketamine was shown to have no effect on brain BDNF in rats, and no correlation was seen between peripheral and central BDNF levels (137). Studies like this contribute to the challenge of interpreting BDNF’s exact role in ketamine’s rapid antidepressant effects.

BDNF acts to modulate several downstream processes essential to ketamine’s treatment efficacy. A protein that is regulated by and downstream to BDNF, is neuropeptide VGF (non-acronym). VGF is an exercise regulated protein that is downregulated by stress and models of depression, and upregulated with exercise (138, 139). VGF levels increase with ketamine administration in the ventromedial prefrontal cortex in mice (140). Inhibition of VGF in the prefrontal cortex decreases ketamine-induced mTOR signaling and inhibits rapid antidepressant-like effects in mice (141). When VGF is artificially overexpressed, behavioral deficits typically caused by chronic restraint stress is prevented (140). When VGF is knocked out, ketamine exposed mice were more susceptible to chronic stress. Thus, it is likely that VGF is one of many proteins utilized by ketamine, via BDNF, to execute its rapid clinical effects.

Beyond BDNF but still within the mTOR signaling pathway is eukaryotic elongation factor 2 kinase (eEF2K), a downstream kinase of mTOR. This kinase is paired with the protein eEF2, an important factor of ribosome translocation of protein synthesis (142). When NMDAR is at its resting state, eEF2K phosphorylates eEF2, which pauses translation of proteins. Thus, NMDAR antagonism stops eEF2 phosphorylation, which promotes translation of transcripts. Administration of ketamine led to rapid decreases in p-eEF2 in the hippocampus, and artificially inhibiting eEF2K led to increased BDNF protein expression (90). In addition, inhibiting eEF2K in BDNF knockout mice had no antidepressant-like effect, further supporting BDNF’s involvement in ketamine’s actions (90). Ketamine administration was shown to increase levels of phosphorylated eEF2K in the prefrontal cortex (143), yet the significance of this result is yet to be clarified, as the location of phosphorylation determines increased or decreased activity and impacts on eEF2 (144).

P70 ribosomal S6 kinase (p70S6K) is another downstream substrate of mTOR that works to promote protein synthesis, and has previously shown decreased levels in depressed subjects compared to healthy controls (145). Ketamine significantly increased levels of p70S6K in primary neuronal cultures, in the mPFC of rats, and NAc, ventral tegmental area, substantia nigra, hippocampus, and basolateral amygdala of mice, with rapamycin abolishing these effects (59, 60, 88, 146, 147). Ketamine also restores levels of p70S6K in mice models of depression. Interestingly, this ketamine-induced increase in p70S6K does not occur in dopamine 3 receptor (D3R) knockout mice (146). This suggests that ketamine’s mechanism of action depends on viable D3Rs (146).

Glycogen synthase kinase 3, or GSK-3, is one of the enzymes in the mTOR signaling pathway responsible for regulating many aspects of the cell cycle such as proliferation and apoptosis (148). This serine/threonine kinase has previously been studied in the context of bipolar disorder, diabetes, and Alzheimer’s disease. In the context of depression, inhibition of this enzyme is required for ketamine’s behavioral antidepressant effect in numerous animal models of depression (149, 150). Constitutively active GSK-3 is associated with resistance to ketamine’s antidepressant effect, and the usage of a GSK-3 inhibitor, lithium, mimics ketamine’s effects (149). Ketamine has shown to decrease levels of GSK-3 in the mouse hippocampus, striata, dentate gyrus, and PFC (109), and may also act through increasing levels of phosphorylated GSK-3β, the inactive form, in rat prefrontal cortex (151, 152). GSK-3 inhibitors act to augment antidepressant-like effects of ketamine when the dose of ketamine is at a sub-threshold value (152).

GSK-3’s activity is a prerequisite for biomolecular changes observed in animal models after ketamine administration. Specifically, ketamine increases membrane levels of the AMPAR subunit, GluA1 (147, 153). However, when GSK-3 is knocked out, ketamine’s effects on GluA1 are abolished, and previously observed increases in BDNF are inhibited (153). Similar results occur when rapamycin is administrated (147). This mechanism may be monitored through postsynaptic density protein 95 (PSD-95), a substrate for GSK-3 that also regulates AMPAR trafficking (153). To add further, ketamine decreases hippocampal membrane levels of phosphorylated-PSD-95, the target of GSK-3 that would promote AMPA-GluA1 internalization (153). Decreased p-PSD-95 would mean more AMPARs would be available on the membrane to be activated. Ketamine also increased PSD-95 in the rat hippocampus (108, 154) and normalized levels of transcription factor Egr-1 (154).

Ketamine’s modulatory actions also converge on to pathways downstream of NMDAR. In a poststroke depression model of rats, ketamine administration significantly improved antidepressant-like symptoms (155). Along with this, there was an upregulation of subtype NMDAR2-β and downregulation of subtype NMDAR2-α, as well as their downstream signaling proteins, β-CaMKII and α-phosphorylation, respectively, in the dentate gyrus (155). Interestingly, no effects on NR1 and NR3 subtypes were seen, suggesting that ketamine is not dependent on all subunits of NMDARs (155).

Decreased levels of a protein upstream of GSK-3, called insulin-like growth factor 2 (IGF2), has previously been implicated in rodent models of depression, with artificial induction of IGF2 expression having protective effects against depressive-like behaviors (156). IGF2 is upregulated with ketamine *in vivo* and *in vitro* (150, 157)—and this requires the inhibition of GSK-3 as well (150). Knocking out IGF2 reduces ketamine’s behavioral antidepressant effect, and mice resilient to induced depression show higher amounts of IGF2 than mice that were susceptible to depression paradigms (150).

There are several limitations to all mTOR results discussed so far. The first limitation is that there seem to be sex-dependent differences in mTOR signaling. Ketamine treatment increased levels of BDNF mRNA in female, not male mice (158), suggesting sex-dependent mechanisms that require both protein translation and transcription. While female rats display greater sensitivity to ketamine at lower doses than male rats, such as at 2.5 mg/kg, this rapid behavioral antidepressant effect was not paired with an increased phosphorylation level of mTOR (159). A similar result was observed in female mice, where there was actually a decrease of mTOR phosphorylation in the prefrontal cortex (32). In addition, ketamine-induced reductions in levels of eEF2K activation in male rats were in agreement with previous results; however, this was not seen in female rats (159). In fact, the increased sensitivity to ketamine was not seen when female rats were ovariectomized—and restored when artificial estrogen and progesterone were administered, suggesting integral roles of gonadal hormones in ketamine’s mechanism of action (159). This gender-specific response to ketamine is not isolated to this one study, as a similar result is seen with acetylation of α-tubulin—another marker of neuroplasticity. Increased acetylation of α-tubulin was observed only in females 24 h post-ketamine administration, suggesting increased stabilization of microtubules (160). These differences may point to possible variations of the mechanism of action or metabolism for ketamine depending on the sex.

The second limitation arises from the fact that these results stem from a combination of different rodent models of depression. Each model may manifest through various phenotypes of depression, and therefore cannot be considered as one consistent valid method of inducing depression. In fact, when a model of resistant depression was utilized, mTOR levels were significantly reduced in the prefrontal cortex, despite a behavioral antidepressant response (161). This suggests that an increase in mTOR levels do not necessarily reflect a behavioral antidepressant response in all cases.

Lastly, the third limitation is observed from a recent study that showed oral rapamycin in humans with depression prolonging ketamine’s antidepressant effect at 2 weeks (162). However, the addition of rapamycin did not affect ketamine’s acute antidepressant effects (162). This study puts preclinical results into perspective and generates questions about the function of mTOR in sustained antidepressant effects. Taken together, while the current field of literature supports mTOR involvement in the mechanism of action for ketamine, these are limitations to consider.

### Inhibition of MAPK/Extracellular Signal-Related Kinases May Be Involved in the Actions of Ketamine

MAPK/ErK signaling pathway is another important pathway involved in neural plasticity, carrying signals from the cell surface to the nucleus. The MAPK pathway regulates CREB, an integral modulator of cell survival. Inhibition of this pathway shows further potentiation of ketamine’s effects in the rat brain. Ketamine increased levels of p-TrkB and pCREB in cultured neurons and the prefrontal cortex, and decreased pCREB in the hippocampus (163, 164). A MAPK inhibitor alone showed similar results and augmented ketamine’s effects when administered together, suggesting that MAPK signaling inhibition may be involved in ketamine’s mechanism (163).

One of the upstream extracellular signaling growth factors that stimulate the MAPK/Erk pathway, among many others, is vascular endothelial growth factor (VEGF), which has previously been discussed as a potential target for treating depression (165). It is an important mitogen that functions in the survival of endothelial cells, neurons, as well as maintenance of synaptic transmission (165), and has previously shown to mimic actions of antidepressants in behavioral models of rodents (166). Neuron-specific deletion of VEGF or its receptor, or the usage of a VEGF neutralizing antibody resulted in ketamine’s behavioral and synaptogenic effects being abolished in rodents (167, 168). Additionally, infusion of VEGF alone into the mPFC produced rapid antidepressant-like effects in these animal models (167, 168). However, in the rat hippocampus, ketamine administration decreased levels of VEGF after 2 h (169). Data from clinical studies show contradictory results. One found no effects in plasma VEGF levels after treatment with ketamine (131), while another found increased plasma levels of VEGFA mRNA and VEGFA protein (170). Ratio of the mRNA of VEGF to pigment epithelial-derived factor (PEDF), a signaling molecule closely linked to VEGF, was increased with ketamine as well (170). This may suggest VEGF as a part of ketamine’s mechanism.

### Regulation of MicroRNA Expression Is Involved in Ketamine’s Mechanism of Action

MicroRNAs (or miRNAs) are non-coding small molecules that are integral in synthesizing proteins, and modulators of protein expression. Members of the MiR-29 family have shown to regulate neuropathological processes relevant to neuroplasticity. Metabotropic glutamate receptor 4 (GRM4), which is predicted to be a target of MiR-29, regulates many major neurotransmitters such as dopamine and glutamate. Inducing depression in rat models was paired with downregulation of miR-29b-3p and increased levels of GRM4 in the prefrontal cortex, which were restored by ketamine administration (171). In fact, pre-treatment of ketamine prevented upregulation of GRM4 in the rat prefrontal cortex, and miR-29b-3p overexpression was followed by a relief of behavioral depressive symptoms (171). An miRNA from a different family, miR-206, was down-regulated in ketamine-treated rodents, which was determined to be a critical regulator of BDNF protein expression (172). In addition, overexpression of miR-206 in primary cultured hippocampal pyramidal neurons impaired ketamine-induced upregulation of neuronal BDNF protein expression (172). Rats vulnerable to chronic mild stress exhibited reduced levels of another microRNA, miR-9-5p, in the hippocampus, which was reversed by ketamine administration in 24 h (173). An *in vitro* model of stress also showed restored levels of miR-9-5p with ketamine (173). These results suggest that the pathway involving microRNAs may significantly be involved in ketamine’s antidepressant mechanism of action.

### Ketamine’s Mechanisms of Action in Bipolar Depression

Ketamine’s rapid antidepressant potential in unipolar depression have initiated the exploration of its treatment effects in bipolar disorder (BD) as well (174–180). However, evidence supporting ketamine’s effects for BD is limited, and the number of randomized controlled trials is significantly less than those exploring unipolar depression. In addition, extra caution is required due to relevant ketamine’s psychomimetic and dissociative effects (181). Still, current evidence seems to support acute ketamine’s rapid antidepressant and anti-suicidal effects in bipolar depression (182–185), though the data that measures the sustainability of this effect is limited. Studies that differentiate BD-I and BD-II is even fewer, though Zarate et al. (178) suggests that results may be similar in both subtypes.

According to our literature search, there are very few studies that investigate ketamine’s mechanism of action for bipolar disorder specifically. Assumptions can be made from mechanistic actions of lithium, a common treatment option for BD, as both ketamine and lithium inhibit GSK-3 (153, 186) in animal models. This suggests glutamatergic modulation as one of the mechanisms employed by ketamine. A clinical study, Lally et al. (187) found that ketamine improved anhedonia, independent from mood, in patients with bipolar depression, and this effect was significantly associated with increased glucose metabolism in the dorsal anterior cingulate cortex and putamen. Interestingly, patients who were taking concomitant lithium, but not valproate, had greater hedonic improvements (187). BDNF as a molecular marker has been robustly linked with bipolar episodes in patients, with low levels of BDNF associated with depressive and manic episodes, as well as their severity (188–190). BDNF upregulation after ketamine administration may partially explain ketamine’s mechanism in BD (191). Patients with euthymic BD have reported decreased expression of HDAC genes compared to control (192), and ketamine has shown to alter levels of HDAC in animal models as well (109, 193), suggesting the involvement of epigenetic mechanisms of ketamine.

## Conclusion

The present systematic review summarized the wide-ranging scope of current literature regarding molecular neuroplastic changes that occur rapidly after exposure to ketamine. We found that these changes in molecular neuroplasticity revolving glutamate, AMPA receptors (AMPAR), mTOR, BDNF/TrkB, VGF, eEF2K, p70S6K, GSK-3, IGF2, Erk, and microRNAs may likely be responsible in mediating and producing ketamine’s rapid antidepressant effects in MDD and bipolar disorder depression. We found studies that report similar findings post ketamine, such as increased glutamate/glutamine cycling, enhanced actions of AMPA receptors, increased activation of mTOR, BDNF/TrkB, and associated signaling molecules. However, no matter how robust a field of evidence is, such as mTOR’s involvement pathway, potential inconsistencies still exist, such as sex-dependent results and differences in sources of tissue in animal models vs. clinical studies. In addition, these studies employ a range of methodologies in various lab environments, models of depression, and doses of ketamine. It is most probable that many different pathways contribute to ketamine’s actions, and it is currently difficult to narrow down which mechanisms are most dominant over others to produce this effect, due to the widespread nature of ongoing research. Further studies should focus on reproducing currently available data with different animal models of depression, larger sample sizes, and long-term studies exploring mechanisms of maintenance and sustained effects to strengthen the evidence.

## Data Availability Statement

The original results presented in the study are included in the article, and further inquiries can be directed to the corresponding author/s.

## Author Contributions

MJYK: acquisition, analysis, interpretation of data for the writing of original manuscript, and writing original manuscript. EH: substantial contributions to the conception and design, and revising it critically for important intellectual content. GV: substantial contributions to the conception or design and final approval of the version to be published. All authors agreed to be accountable for all aspects of the work in ensuring that questions related to the accuracy or integrity of any part of the work are appropriately investigated and resolved.

## Conflict of Interest

The authors declare that the research was conducted in the absence of any commercial or financial relationships that could be construed as a potential conflict of interest.

## Publisher’s Note

All claims expressed in this article are solely those of the authors and do not necessarily represent those of their affiliated organizations, or those of the publisher, the editors and the reviewers. Any product that may be evaluated in this article, or claim that may be made by its manufacturer, is not guaranteed or endorsed by the publisher.
